# Susceptibility to COVID-19 and Immunologic Response to Vaccination in Patients With Immune-Mediated Inflammatory Diseases

**DOI:** 10.1093/infdis/jiad148

**Published:** 2023-08-04

**Authors:** Axel Finckh, Adrian Ciurea, Catherine E Raptis, Andrea Rubbert-Roth

**Affiliations:** Division of Rheumatology, Geneva University Hospitals, Geneva, Switzerland; Geneva Centre for Inflammation Research, Faculty of Medicine, University of Geneva, Geneva, Switzerland; Department of Rheumatology, Zurich University Hospital, University of Zurich, Zurich, Switzerland; Swiss Clinical Quality Management Foundation, Zurich, Switzerland; Division of Rheumatology and Immunology, St. Gallen Cantonal Hospital, St. Gallen, Switzerland

**Keywords:** COVID-19, immune-mediated inflammatory disease, mRNA, musculoskeletal, rheumatoid, vaccination, vaccine, SARS-CoV-2

## Abstract

Immune-mediated inflammatory diseases (IMIDs) are a highly heterogeneous group of diseases that share a common etiology of immune dysregulation, such as rheumatoid arthritis, inflammatory bowel disease, and psoriasis, among others. It is estimated that the prevalence of IMIDs ranges between 5% and 7% in developed countries. As current management of IMIDs includes the use of immunomodulatory medications, the resulting weakened immune response can increase the risk of infection, including with SARS-CoV-2 (the causative agent of COVID-19) and reduce response to vaccination, placing these individuals at continued risk of severe outcomes from COVID-19. In this article, we summarize the current literature related to COVID-19 outcomes and the immunogenicity and reactogenicity of COVID-19 mRNA vaccination among patients with rheumatologically dominated IMIDs, as well as the effect of immunomodulatory therapies on these outcomes. We conclude by providing current COVID-19 vaccination recommendations for individuals with IMID.

## PATIENTS WITH IMMUNE-MEDIATED INFLAMMATORY DISEASES ARE AT ELEVATED RISK OF COVID-19

Immune-mediated inflammatory diseases (IMIDs) are a highly heterogeneous group of diseases that share a common etiology of immune dysregulation [1, 2]. IMIDs together have an estimated prevalence of 5% to 7% in developed countries [3] and include, but are not limited to, those primarily affecting the joints/spine (rheumatoid arthritis [RA], spondyloarthritis, psoriatic arthritis), intestinal tract (inflammatory bowel disease: Crohn's disease, ulcerative colitis), skin (psoriasis, atopic dermatitis), and connective tissues (systemic lupus erythematosus, systemic sclerosis) [1, 2, 4]. Patients with IMID have a chronic disease course and often have alternating periods of relatively low disease activity and periods of disease exacerbation or flaring [4]. Current management of IMIDs includes the use of immunomodulatory medications, with a wide range of immune system targets, which are employed depending on disease activity and organ involvement [1, 4, 5].

The weakened immune response caused by immunomodulatory medications in patients with IMIDs can increase the risk of infection, including with SARS-CoV-2, the causative agent of COVID-19 [6, 7]. Vaccination with the safe and effective COVID-19 mRNA vaccines, such as mRNA-1273 and BNT162b2, is the primary strategy for the prevention of COVID-19 [5]; however, the immunosuppressive therapies that patients with IMIDs are treated with may result in a reduced response to vaccination [8] and place them at continued risk of severe outcomes from COVID-19.

While COVID-19 mitigation strategies for the IMID population include prioritization for vaccination against COVID-19, the complexity of treatment in this patient population and suboptimal immune responses following vaccination remain a challenge for healthcare professionals. Notably, as the COVID-19 pandemic continues and potentially moves into a more endemic state, a thorough understanding of vaccine responses among people with IMIDs, including those treated with immunosuppressive therapy, is crucial to inform clinical practice. In this article, we summarize the current literature related to COVID-19 outcomes and the immunogenicity and reactogenicity of COVID-19 mRNA vaccination among patients with IMIDs, as well as the effect of immunosuppressive therapies on these outcomes. We primarily focus on findings from patients with rheumatologically dominated IMIDs.

## BURDEN OF DISEASE FOR COVID-19 IN THE IMID POPULATION

### Susceptibility to SARS-CoV-2 Infection and COVID-19 Symptom Severity and Duration

Higher rates of SARS-CoV-2 infection and COVID-19–related hospitalization and death have been reported among patients with IMIDs, especially before the widespread availability of COVID-19 vaccines [9, 10]. A retrospective matched cohort study based on records (between January 20 and August 15, 2020) from a US multicenter electronic health network noted a higher risk of hospitalization (relative risk [RR], 1.14; 95% confidence interval [CI], 1.03–1.26) and intensive care unit admission (RR, 1.32; 95% CI, 1.03–1.68) among COVID-19 patients with IMIDs relative to patients without IMIDs [10]. Furthermore, a large retrospective, matched cohort study of US veterans (between January 1 and December 10, 2020) showed that the risk of SARS-CoV-2 infection was 25% higher (hazard ratio [HR], 1.25; 95% CI, 1.13–1.39) and the risk of severe COVID-19 symptoms (resulting in hospitalization/death) was 35% higher (HR, 1.35; 95% CI, 1.10–1.66) among individuals with RA in comparison to individuals without RA [9].

In addition to the increased susceptibility to and severity of COVID-19 among patients with IMIDs, studies have shown that COVID-19 symptoms may persist for longer in comparison with the general population. Prolonged COVID-19 symptom duration has been reported in a global survey of patients with IMIDs conducted between April 2 and October 15, 2021 (following COVID-19 vaccine availability in some geographic regions) [11]. Among the 441 individuals with IMIDs surveyed, COVID-19 symptoms were present for a median duration of 15 days versus 7 to 12 days for the general population [11]. COVID-19 symptom duration was ≥28 days in 24% (107/441) and ≥90 days in 10% (42/441) of surveyed patients with IMIDs [11]. The influence of immunomodulating medications on COVID-19 symptom duration was not examined in this study but was identified as a factor potentially impacting the risk for prolonged COVID-19 symptom duration [11]. Therefore, among the population with IMIDs, there is increased susceptibility to SARS-CoV-2 infection, as well as increased COVID-19 symptom severity and duration.

### Immunosuppressive Medications and Impact on COVID-19 Outcomes in Patients With IMIDs

The immunosuppressive medications taken by patients with IMIDs, rather than the disease itself, may be the primary contributor to poor outcomes following COVID-19 in this population [9, 12]. Specifically, in a retrospective matched cohort study of US veterans with RA between January 1 and December 10, 2020 (before availability of COVID-19 vaccination), those who received treatment with glucocorticoids and disease-modifying antirheumatic drugs (DMARDs) had the highest risk of SARS-CoV-2 infection and COVID-19–related hospitalization or death [9]. The risk of SARS-CoV-2 infection was 66% higher (HR, 1.66; 95% CI, 1.36–2.03) and the risk of COVID-19–related hospitalization or death was 112% higher (HR, 2.12; 95% CI, 1.48–3.03) among veterans with RA who were treated with prednisone and biologic and targeted synthetic DMARDs relative to individuals without RA [9]. The increased risk of severe COVID-19 in patients with IMIDs has been linked to certain immunosuppressive therapies, such as rituximab, Janus kinase (JAK) inhibitors, and glucocorticoids [12–14].

Patients with IMIDs are concerned about flares of their underlying disease after acute COVID-19. However, in certain cases the IMID flaring may be caused by immunosuppressive medication disruption rather than by COVID-19 itself. A prospective study in the United States examined 174 patients with IMIDs who survived COVID-19 between March 1, 2020 and November 3, 2021 (a minority [9%] of patients were vaccinated before infection), and found that 41% experienced an IMID flare after COVID-19 [15]. The time between COVID-19 and the IMID flare was <1 week in 20%, between 1 and 4 weeks in 44%, and between 4 and 12 weeks in 27% of patients [15]. Medication disruption at COVID-19 onset likely contributed to the IMID flaring observed in this study; among patients who were prescribed DMARDs, 51% reported changes to their use at COVID-19 onset, including temporarily stopping, lengthening the dosing interval, decreasing the dose, and starting a new DMARD [15].

Therefore, the prescribed use of immunomodulatory therapy, as well as the altered use of medication during an episode of COVID-19, rather than the disease itself, may be the primary contributor to poor outcomes following COVID-19. It is important to understand how the disease and disease therapy may impact responses to COVID-19 vaccination or underlying disease flaring in this population to better guide vaccination schedules.

## RESPONSE TO COVID-19 mRNA VACCINATION IN THE IMID POPULATION

### Immunogenicity Following COVID-19 mRNA Vaccination (2-Dose Primary Series)

It is well established that IMID populations respond less favorably to vaccination than healthy adults [16, 17]. Although initial COVID-19 mRNA vaccine trials excluded patients with IMIDs, it quickly became clear that following the recommended 2-dose primary series of COVID-19 mRNA vaccination, serologic response rates were lower among IMID populations than among immunocompetent adults [8, 18]. COVID-19 mRNA vaccine responses among the IMID population are summarized in Table 1. Two systematic reviews and meta-analyses have already examined the immune response after 2 doses in patient populations with IMIDs [8, 18]. Specifically, Jena and colleagues reported that the odds of seroconversion were substantially lower for individuals with IMIDs compared with healthy controls (odds ratio [OR], 0.05; 95% CI, .02–.13); pooled seroresponse rates were 88% among all patients with IMIDs, 80% among the subgroup of patients with RA, and 96% among patients with spondyloarthritis [8]. Similarly, Sakuraba and colleagues reported a pooled seroresponse rate of 83% among all patients with IMIDs and 80% among the subgroup of patients with rheumatic diseases [18]. The odds of seroconversion for patients with rheumatic diseases was lower than for healthy controls (OR, 0.068; 95% CI, .016–.29) [18].

**Table 1.** COVID-19 mRNA Vaccine Responses Among the IMID Population

Study Citation	Study Type	Vaccine(s)	IMID Population	Seropositivity Rates	T-Cell Response Rates
2 Doses					
Jena et al, 2022 [8]	Meta-analysis	mRNA	11 studies of patients with IMIDs (n = 1297), including 7 studies of patients with RA (n = 488) and 3 studies of patients with SpA (n = 101)	88% (95% CI, 81%–92%) Patients with RA: 80% (95% CI, 65%–89%) Patients with SpA: 96% (95% CI, 83%–99%) Patients receiving rituximab: 30% (95% CI, 14%–52%)	Not reported
Sakuraba et al, 2021 [18]	Meta-analysis	mRNA	18 studies of patients with IMIDs (n = 2534), including 7 studies of patients with rheumatic diseases (n = 1702)	83% (95% CI, 77%–88%) Patients with rheumatic diseases: 80% (95% CI, 68%–89%)	Not reported
Deepak et al, 2021 [19]	Prospective cohort study in the United States (COVaRiPAD)	BNT162b2 or mRNA-1273	133 patients with chronic inflammatory diseases: 42 (32%) with IBD, 38 (29%) with RA, 20 (15%) with SpA,^a^ and 33 (25%) with a range of other diseases	89% (118/133) Patients receiving B-cell–depleting therapy: 60% (6/10) Patients receiving glucocorticoids: 65% (11/17)	Not reported
			53 immunocompetent participants	100% (53/53)	Not reported
Siero Santos et al, 2021 [20]	Longitudinal study in Spain	BNT162b2 or mRNA-1273	147 patients with immune-mediated rheumatic diseases: 55 (37%) with RA, 44 (30%) with SLE, 24 (16%) with systemic sclerosis, and 24 (16%) with Sjogren's disease	63% (93/147) Patients without immunosuppression: 80% (38/47) Patients receiving immunosuppressants: 55% (55/100) Patients receiving abatacept: 10% (1/10) Patients receiving rituximab: 31% (5/16) Patients receiving rituximab + methotrexate: 30% (3/10)	CD4: 59% (87/147) CD8: 61% (89/147) Patients receiving immunosuppressants: CD4: 52% (52/100) CD8: 53% (53/100) Patients without immunosuppression: CD4: 75% (35/47) CD8: 77% (36/47) Patients receiving abatacept: CD4: 10% (1/10) CD8: 20% (2/10) Patients receiving rituximab: CD4: 50% (8/16) CD8: 50% (8/16) Patients receiving rituximab + methotrexate: CD4: 50% (5/10) CD8: 50% (5/10)
			50 healthy controls	100% (50/50)	CD4: 100% (50/50) CD8: 92% (46/50)
Syversen et al, 2022 [21]	Prospective study in Norway (Nor-vaC)	BNT162b2, mRNA-1273, or ChAdOx1nCoV-19 (first dose only)	1647 patients with IMIDs (excluding those on CD20-depleting therapy): 475 (29%) with IBD, 566 (34%) with RA, 305 (19%) with SpA, and 295 (18%) with PsA	91% (1504/1647) Patients with RA: 89% (503/566) Patients with SpA: 89% (271/305) Patients with PsA: 97% (286/295) BNT162b2: 89% (1026/1152) mRNA-1273: 98% (391/401) Patients receiving abatacept: 53% (8/15)	Not reported
			1114 healthy controls	98% (1096/1114)	Small subset examined (20/1114): CD4: 100% (20/20) CD8: 100% (20/20)
Jyssum et al, 2022 [22]			87 patients with RA receiving rituximab (CD20-depleting therapy)	22% (19/87)	Small subset examined (20/87): CD4: 53% (10/19) CD8: 74% (14/19)
Kondo et al, 2022 [23]	Prospective study in Japan	BNT162b2 or mRNA-1273	974 patients with inflammatory rheumatic diseases (796 received BNT162b2; 178 received mRNA-1273)	86% (836/974) BNT162b2: 83% (663/796) mRNA-1273: 97% (173/178)	Not reported
			630 healthy controls (all received BNT162b2)	99.5% (627/630)	Not reported
Schreiber et al, 2022 [24]	Prospective study in Denmark (DECODIR)	BNT162b2 or mRNA-1273	243 patients with inflammatory rheumatoid diseases: 142 (58%) with RA, 60 (25%) with PsA, and 39 (16%) with SpA	70% (171/243)	Not reported
3 Doses					
Syversen et al, 2022 [21]	Prospective study in Norway (Nor-vaC)	BNT162b2, mRNA-1273, or ChAdOx1nCoV-19 (first dose only)	153 IMID patients with weak serologic response >3 wk after 2 doses: 52 (34%) with RA, 21 (14%) with PsA, 16 (10%) patients with SpA, and 64 (42%) with IBD	94% (129/153)	Not reported
Jyssum et al, 2022 [22]			49 patients with RA receiving rituximab (CD20-depleting therapy) with weak serologic response >3 wk after 2 doses	16% (8/49) and a weak response observed in an additional 25% (12/49)	Small subset examined: CD4: 100% (12/12) CD8: 100% (12/12)
Azzolini et al, 2022 [25]	Observational study in Italy	mRNA-1273	48 patients with rheumatic diseases	>90% (numbers not reported)	Not reported
Kim et al, 2022 [26]	Observational cohort study in Korea	BNT162b2 or mRNA-1273	57 patients with autoimmune rheumatic diseases	47% (27/57) had omicron neutralization	Not reported
Kartnig et al, 2022 [27]	Prospective study in Austria	BNT162b2 or mRNA-1273	56 patients with IMIDs (excluding those on CD20-depleting therapy), including 25 (45%) with inflammatory arthritis	91% (51/56)	Not reported
			47 healthy controls	100% (47/47)	Not reported
4–5 Doses					
Teles et al, 2022 [28]	Prospective observational study	BNT162b2 or mRNA-1273	16 patients with autoimmune diseases	100% (16/16)	Not reported

Abbreviations: CI, confidence interval; IBD, inflammatory bowel disease (Crohn's disease or ulcerative colitis); IMID, immune-mediated inflammatory disease; PsA, psoriatic arthritis; RA, rheumatoid arthritis; SLE, systemic lupus erythematosus; SpA, spondyloarthropathy.

Axial spondyloarthritis, psoriatic arthritic/psoriasis, or IBD-associated arthritis.

Additional prospective or longitudinal observational studies have examined the immune response to a 2-dose primary series of COVID-19 mRNA vaccination among populations with IMIDs. These studies also found reduced seroconversion rates among the IMID populations (range, 63%–91%) compared with healthy controls (range, 98%–100%) [19–21, 23, 24]. In addition to antibody responses, cellular immune responses are believed to play an important role in protection against COVID-19 [20]. A study found reduced cellular responses following COVID-19 vaccination among the IMID population versus healthy controls [20]. While 100% and 92% of healthy controls had CD4 and CD8 T-cell responses, respectively, in contrast, only 59% and 61% of the IMID population had CD4 and CD8 T-cell responses [20].

The type of COVID-19 mRNA vaccine received may also influence immunogenicity among IMID populations. Receipt of 2 doses of mRNA-1273 (as compared with BNT162b2) was found to be a significant predictor of serologic response (multivariate OR, 4.45; 95% CI, 1.66–11.92; *P* = .002) among 1647 patients with IMIDs in a large prospective observational study [21]. Serologic response was achieved by 98% of patients who received mRNA-1273 versus 89% who received BNT162b2 [21]. Another prospective study noted similar results, with seroconversion rates of 97% for recipients of mRNA-1273 versus 83% for BNT162b [23].

Together, these studies suggest that following the 2-dose primary series of COVID-19 mRNA vaccination, serologic response rates are lower among IMID populations than healthy controls and that rates may vary by the type of mRNA vaccine received. Rather than the disease itself impacting responses, it is likely to be the type of immunosuppressive medication that is the primary contributor to a reduced immune response following a 2-dose primary vaccination series with an mRNA vaccine [29]. Of note, among individuals with IMIDs, seroconversion rates are lower among those administered immunosuppressive therapies (55%) compared with those without immunosuppression (80%) [20]. The use of DMARDs has been demonstrated to reduce the immune response 6 weeks after initial vaccination in a prospective cohort study [24]. Pausing methotrexate therapy following at least 1 of the 2 primary doses has been shown to increase immunogenicity [30]. A recent meta-analysis demonstrated that patient populations with IMIDs receiving abatacept or anti-CD20 B-cell–depleting therapy (rituximab) had seroconversion rates of <70% following a 2-dose primary series of mRNA vaccine; patients receiving steroids, hydroxychloroquine, methotrexate, JAK inhibitors, belimumab, leflunomide, or mycophenolate mofetil had seroconversion rates of 70% to 90%; and patients receiving anti-integrin, anti-tumor necrosis factor (TNF), anti-interleukin 17 (IL-17), anti–IL-6, ustekinumab, or aminosalicylates (5-ASA) had seroconversion rates of >90% [8]. Prospective studies conducted following publication of the aforementioned meta-analysis confirmed the finding that abatacept or rituximab/B-cell–depleting therapy were associated with seroconversion rates of <70% [19–21].

A prospective study specifically examined immune responses among 87 patients with RA who were receiving rituximab [22]. Serologic response after the 2-dose primary series was achieved by only 22% of patients [22]. Time between the last rituximab infusion and the first vaccine dose was found to be a significant predictor of serologic response (multivariate OR, 2.97; 95% CI, 1.67–5.29; *P* = .0002) [22]. The study found that the median interval between rituximab and the first vaccine dose was 9 months among serologic responders compared with only 4 months among nonresponders [22]. Based on these findings, the authors concluded that the interval between rituximab infusion and vaccination should be as long as possible to optimize serologic response, preferably at least 9 months [22]. However, time since last rituximab infusion was not correlated with T-cell response [22]. T-cell responses were detected among all healthy controls examined, whereas CD4 and CD8 T-cell responses were detected in 53% and 74% of patients with RA, respectively [22]. These findings were consistent with another study of patients receiving anti-CD20 treatment with rituximab or ocrelizumab [31]. Therefore, some patients with RA receiving rituximab may achieve cellular immune responses following vaccination in the absence of serologic response, which could offer some protection from COVID-19. Antibody response to COVID-19 mRNA vaccination among patients receiving anti-CD20 therapy seems to be correlated with the number of circulating CD19^+^ B cells measured at day 30 following vaccination, which may be a useful biomarker for clinicians evaluating when to administer additional vaccine doses to these patients [31].

In conclusion, immunosuppressive therapies dampen the immune response to varying degrees following mRNA vaccination against COVID-19 in individuals with IMIDs. Treatment with rituximab (anti-CD20 B-cell–depleting therapy) and abatacept (costimulation blockade) is associated with the lowest seroconversion rates, whereas anti-cytokine therapies, such as anti-integrin, anti-TNF, anti–IL-17, anti–IL-6, anti–IL-23, or 5-ASA therapies, had the least effect on seroconversion rates.

### Durability of Immune Response to COVID-19 mRNA Vaccination (2-Dose Primary Series)

Durability is an important component of assessing vaccine-induced immunogenicity and long-term protection against COVID-19, which is especially relevant in this population as the circulation of the virus becomes more endemic. As discussed previously, individuals with IMID generally mount poorer serologic responses following a 2-dose COVID-19 mRNA vaccination series compared with healthy individuals, but it is unclear whether there are differences in durability of response. In a prospective study in Germany, 2535 patients with IMIDs and 1198 healthy controls were followed up for 40 weeks after the first dose; reduced intensity and longevity of the antibody response following 2 doses of COVID-19 vaccination were observed among patients with IMIDs [32]. A prospective observational study in Hungary examined 89 patients with IMIDs and 74 healthy controls who received COVID-19 vaccination [33]. Four months following the second dose of mRNA vaccine, the SARS-CoV-2 neutralizing anti-receptor binding domain antibody levels in the IMID population were only 81% compared with healthy controls [33], indicating adequate durability but at levels lower than the general population. As expected, 1 and 4 months following vaccination, patients receiving B-cell inhibitory treatment had lower antibody levels than patients receiving other biologic DMARDs [33]. In a recent UK single-cohort study of rituximab-treated patients with IMIDs, among individuals vaccinated against COVID-19, the rates of severe COVID-19 (hospitalization/death) from SARS-CoV-2 breakthrough infections were comparable to the rates of severe infections seen with viruses other than SARS-CoV-2 [34]. The study authors concluded that rituximab therapy may still be favored among vaccinated patients with severe rheumatic or musculoskeletal diseases if few other treatment options exist [34].

Similar to primary immune responses, the type of COVID-19 mRNA vaccine received may influence the durability of immune response following vaccination among IMID populations. Results from a US study of 326 patients with IMIDs showed that 96% of patients had positive anti-spike antibody titers at 1 month and 6 months after the second dose, indicating good durability of response [35]. More patients who received mRNA-1273 (89%) compared with BNT162b2 (72%) had high antibody titers at 6 months [35]. In agreement with these findings, results from an observational study of 565 patients with inflammatory rheumatic diseases in Switzerland noted significantly higher odds of increased anti-spike antibody levels at week 24 following dose 2 of mRNA-1273 versus BNT162b2 (OR, 3.8; 95% CI, 2.7–5.2; *P* < .0001) [36]. This difference was even more striking in elderly patients [36]. A US observational cohort study found that there were more COVID-19 breakthrough infections among patients who received BNT162b2 (n = 446) than mRNA-1273 (n = 329) over a mean follow-up of 11 to 12 months [37]. When follow-up was limited to the pre-omicron era, more breakthrough infection events were observed with BNT162b2 (n = 158) versus mRNA-1273 (n = 94), although the differences were not statistically significant [37].

### Approaches to Enhancing the Immune Response

The initial recommendation for patients with IMIDs was to receive a 2-dose primary vaccination schedule of mRNA vaccines; however, it soon became evident that 2 doses may not be sufficient in this population. As a result, additional vaccination strategies were assessed, including a 3-dose primary series and additional booster doses. The American College of Rheumatology (ACR) and European Alliance of Associations for Rheumatology (EULAR) currently recommend the administration of supplemental vaccine doses to induce stronger immune responses in IMID populations [5, 38]. In addition, pausing specific immunomodulatory medications around vaccinations in patients with stable IMIDs may be recommended.

Higher vaccine-induced neutralizing antibody levels have been associated with more profound protection against symptomatic infection [39]. Although monitoring of vaccine-induced immune responses is not routinely recommended [5], it may guide the management of individual patients by identifying those who would benefit from receiving an earlier third vaccine dose [40]. Results from a prospective cohort study in Switzerland noted lower humoral immune responses at 2 weeks following the 2-dose primary series in patients with RA receiving various DMARD regimens, with antibody levels at 2 weeks following the second dose predicting the neutralizing antibody activity at 24 weeks [40]. Analyzing isotypic antibody responses to various viral antigens [41] revealed that vaccine-induced immunoglobulin (Ig) G responses were diminished, while IgA and IgM responses persisted, indicating that a delayed isotype switch may underlie the delay in mounting strong IgG responses [40]. However, it is important to remind patients that antibody levels only measure part of the immune response to vaccination.

#### Adoption of 3-Dose Primary Series for COVID-19 mRNA Vaccines

To address the inadequate immune response in patients with IMIDs receiving any immunomodulatory therapy after 2 mRNA vaccine doses, the addition of a third primary dose at least 28 days after the second dose was recommended by the Centers for Disease Control and Prevention. Observational data have supported an additional primary dose in this population (Table 1). Specifically, a study in Italy that included patients with rheumatic disease with moderate to high levels of immunosuppression showed a substantial boost in IgG serum levels and neutralization capacity 2 weeks following the third mRNA-1273 dose in comparison to levels observed 2 to 4 months after the second vaccine dose [25]. A prospective cohort study in Switzerland administered an early third mRNA vaccine dose in patients with RA who did not mount an antibody response within 12 weeks of the second dose; DMARD therapy was paused for 2 weeks before and after the third dose. This strategy resulted in a substantial antibody response in the majority of patients [42]. Pausing methotrexate treatment for 2 weeks following the third vaccine dose has also been shown to boost the antibody response among patients with IMIDs in the United Kingdom [43]. The benefit of a third dose was also noted in a prospective study in Norway, where 94% of patients had a serologic response following the third dose in an IMID population, with an inadequate serologic response to the 2-dose primary series [21]. A separate analysis from this study examined 49 patients with RA receiving rituximab (CD20-depleting therapy); 8 had a serologic response, 12 had a weak response, and 29 had no response [22]. CD4 and CD8 T-cell responses were detectable in all 12 patients examined [22]. Therefore, evidence suggests that rituximab is associated with low seroconversion rates following a third vaccine dose; however, cellular immune responses do not appear to be negatively impacted.

#### Emerging Variants of Concern and Booster Doses

Variant-updated booster vaccines may expand immune protection against emergent SARS-CoV-2 variants of concern, such as omicron and its subvariants, in the IMID population. While the third dose of mRNA vaccine is immunogenic in the majority of the IMID population, the neutralizing antibody responses against the omicron variant appear to be lower in comparison with wild-type SARS-CoV-2. Results from an observational cohort study in Korea showed that 47% of patients with autoimmune rheumatic diseases had omicron-neutralization responses following receipt of an mRNA vaccine as a third dose [26]. In a prospective clinical trial in Austria of 56 patients with IMIDs and 47 healthy controls who received a third dose of mRNA vaccine, median neutralizing antibodies against wild-type SARS-CoV-2 and omicron BA2 were approximately 2-fold higher in healthy controls compared with patients with IMIDs [27]. Patients treated with combination DMARD therapy (conventional synthetic DMARD + biologic DMARD) had lower neutralizing antibody responses against the wild-type virus and omicron BA2 than patients not receiving these therapies [27]. An observational study found that 26 patients who paused methotrexate therapy after receipt of the third mRNA vaccine dose had increased neutralizing capacity against omicron BA1 and BA2 to levels comparable to 44 nonimmunosuppressed individuals at week 4 [44].

In a Norwegian prospective observational study of patients with IMIDs under immunosuppression versus healthcare worker controls, patients who received a fourth mRNA vaccine booster dose (at a median of 106 days after third dose) had higher antibody levels than after the third dose, but lower in comparison with levels observed in the control group after a third vaccine dose [45]. Additionally, patients with COVID-19 infection (natural infection) after the third vaccine dose had higher antibody concentrations than patients with a fourth vaccine dose [45]. In another prospective observational study, 16 adults with autoimmune diseases reported receiving an initial 3-dose primary vaccination series against COVID-19, followed by 2 additional booster doses (fifth dose at a median of 126 days after fourth dose); 6 patients were seronegative after the first 2 doses, 2 were seronegative following dose 3, and 1 was seronegative following dose 4 [28]. Furthermore, all patients were seropositive for SARS-CoV-2 antibodies at a median of 26 days after dose 5 [28]; however, some patients had a response below the threshold of the proposed minimum level required for neutralization against omicron [28].

Based on these findings, it appears that patients with IMIDs with and without an adequate response to the 2-dose primary series can derive immunologic benefit from additional vaccine doses; however, immunosuppressive medications may dampen the serologic response and the neutralization capacity against the omicron variant may be lower. Future studies are needed to examine whether delays in IMID therapy or vaccine doses can optimize immune responses to COVID-19 vaccination. Alongside COVID-19 vaccination, the recent initiation of outpatient antiviral and monoclonal antibody treatment has significantly lowered the odds of severe COVID-19 outcomes (hospitalization/death, OR, 0.12; 95% CI, .05–.25) versus no outpatient treatment in a retrospective cohort study of 704 patients with IMIDs who had COVID-19 onset between January and May 2022 [46]. Among the 426 patients who received outpatient treatment for COVID-19, 72% (307/426) received nirmatrelvir (an oral protease inhibitor) in combination with ritonavir (a pharmacokinetic boosting agent) and 25% (105/426) received monoclonal antibodies [46]. Both antiviral treatment and monoclonal antibodies were associated with lower odds of COVID-19 hospitalization or death compared with no outpatient treatment (nirmatrelvir-ritonavir, OR, 0.08, 95% CI, .03–.24; monoclonal antibodies, OR, 0.20, 95% CI, .07–.54) [46]. Additionally, a small, single-center study examined whether preexposure prophylaxis with anti–SARS-CoV-2 monoclonal antibodies (tixagevimab and cilgavimab) provided benefit to severely immunocompromised patients with IMIDs who did not generate an adequate response to mRNA vaccination (4 doses) [47]. Of 17 eligible patients, 10 received preexposure prophylaxis with anti–SARS-CoV-2 monoclonal antibodies [47]. Of these 17 patients, 8 subsequently had PCR-confirmed COVID-19, of whom 87.5% (7/8) had not received the preexposure prophylactic anti–SARS-CoV-2 monoclonal antibodies; the 1 patient who did receive preexposure prophylactic monoclonal antibodies had a mild disease course and did not require hospitalization [47]. Of note, detailed discussion of antiviral treatments and monoclonal antibody prophylaxis is beyond the scope of this review. No general recommendation on the use of therapeutic antibodies can be made and their use should be applied according to regional recommendations and currently circulating SARS-CoV-2 variants.

## SAFETY AND REACTOGENICITY OF COVID-19 mRNA VACCINATION IN THE IMID POPULATION

Patients with IMIDs experience the same range of adverse events as healthy controls following COVID-19 mRNA vaccination, but these events are less common in the IMID population than in immunocompetent individuals [21]. Following the 2-dose primary series of COVID-19 mRNA vaccination in a longitudinal observational study in Norway, adverse events were reported by 78% of healthy controls (191/244) and 53% of patients with IMIDs (810/1516) [21]. After dose 3, adverse events were reported by 44% of patients (70/159); the most common adverse events in patients after doses 2 and 3 included pain at the injection site, slackness, headache, and tiredness [21]. Based on the study findings, the authors concluded that the lower frequency of adverse events among patients with IMIDs could be attributed to the immunosuppressive medications [21]. The most common immunosuppressive medications in the study were TNF inhibitors and methotrexate monotherapy [21]. It is unclear whether there was a correlation between patients with reduced immunogenicity and reactogenicity following mRNA vaccination, which has been previously proposed [48].

There is some concern regarding the potential risk of underlying IMID flaring following COVID-19 vaccination [5]. However, IMID flaring observed in some patients following COVID-19 mRNA vaccination may have been caused by patients pausing their immunosuppressive medications rather than by the vaccine itself [21]. Of note, in this same study, disease flares were reported by 6% of patients following dose 2 and 16% following dose 3 [21]; all patients who experienced a disease flare following dose 3 had inflammatory joint disease [21]. The study authors concluded that disease flaring following dose 3 may have been caused by the study requirement for patients with inflammatory joint disease to pause their medication from 1 week before until 2 weeks after the third vaccine dose [21].

## COVID-19 VACCINATION RECOMMENDATIONS FOR THE IMID POPULATION

The ACR and EULAR recommend the administration of supplemental mRNA vaccine doses following the 3-dose primary series to induce stronger immune responses in the IMID population and to consider altering the timing of some medications, such as rituximab and abatacept, to optimize immunogenicity (Table 2) [5, 38]. Studies have shown that pausing DMARDs for a short period (eg, methotrexate for 2 weeks) increases the effectiveness of booster vaccination among patients with IMIDs, without increasing the risk of disease flares at week 12 [43]. However, evidence defining the optimal timing of vaccination with respect to rituximab is lacking [38]. The benefit of vaccination against COVID-19 is considered to outweigh the potential risk of underlying disease flaring [5]. Ultimately, healthcare professionals should involve their patients with IMIDs in a shared decision-making process regarding COVID-19 vaccination [5, 38].

**Table 2.** COVID-19 mRNA Vaccination Recommendations for Patients With IMIDs Affecting the Joints/Spine

Society	Guidance Document	Considerations/Recommendations
American College of Rheumatology	American College of Rheumatology Guidance for COVID-19 Vaccination in Patients With Rheumatic and Musculoskeletal Diseases: Version 5. 2023 [5]	Primary vaccination, supplemental dosing, and booster doses should be given regardless of whether patients have experienced a natural infection Patients who received 3 doses of vaccine (primary series)^a^ and who are expected to have mounted an inadequate vaccine response should receive supplemental doses (eg, ≥2 additional boosters, for a total of 5 doses) as recommended by the CDC for immunocompromised individuals For both the Moderna and Pfizer mRNA vaccines, a booster shot is recommended at ≥3–4 mo after completion of the 3-dose primary vaccine series The benefit of vaccination outweighs the potential risk of underlying disease flaring Patients should receive vaccination, consistent with the age restriction of the regulatory approval, and should be prioritized before the general population of similar age and sex The expected response to vaccination among many patients receiving systemic immunomodulatory therapies is blunted in its magnitude and duration compared with the general population Timing considerations for immunomodulatory therapy in relation to vaccination are provided in the guidance document, including for abatacept^b^ and rituximab^c^
European Alliance of Associations for Rheumatology	EULAR recommendations for the management and vaccination of people with rheumatic and musculoskeletal diseases in the context of SARS-CoV-2: the November 2021 update. 2022 [38]	Patients should be advised to receive vaccination with any approved vaccine and booster/supplemental doses as recommended by several authorities For patients not currently taking immunomodulatory or immunosuppressive treatment, vaccination should precede a treatment start if clinically feasible For patients using rituximab or another B-cell–depleting therapy, vaccination should be scheduled to optimize immunogenicity. Postponing the next cycle of rituximab should be considered. However, there is a lack of evidence to define optimal timing of vaccination with respect to rituximab

Abbreviations: CDC, Centers for Disease Control and Prevention; EULAR, European League Against Rheumatism; IMID, immune-mediated inflammatory disease.

Third dose should be administered at least 28 days after the second dose to complete the primary series.

Abatacept: patients receiving subcutaneous therapy are recommended to pause abatacept for 1 week before and 1 week following each vaccination dose; patients receiving intravenous infusion are recommended to time each vaccination dose at 4 weeks after the last infusion and to postpone the subsequent infusion by 1 week [5].

Rituximab: schedule the start of vaccination series approximately 4 weeks before the next scheduled rituximab cycle and after the final vaccine dose to delay rituximab by 2–4 weeks, if disease activity allows [5].

## CONCLUDING REMARKS

Patients with IMIDs are at higher risk for severe outcomes from COVID-19 compared with the general population; this risk may be caused by their IMID therapies rather than the IMID itself. Patients with IMIDs receive a wide range of immunomodulatory treatments, which can weaken their immune responses to infections. Patients with IMIDs who are receiving systemic immunomodulatory therapies are expected to have a blunted immunologic response to mRNA vaccination in terms of both magnitude and duration compared with the general population [5]. Even though the immune responses to COVID-19 vaccination are dampened among patients with IMIDs in comparison with the general population, the vaccine is still expected to provide protection against severe outcomes, and patients with IMIDs should be prioritized for vaccination [5]. Additional studies including larger population sizes and those who received bivalent booster doses are needed to inform ongoing vaccination strategies for this population to ensure they are optimally protected from COVID-19.

## Notes

***Acknowledgments.*** Medical writing and editorial assistance were provided by Renee Gordon, PhD, of MEDiSTRAVA in accordance with Good Publication Practice (GPP 2022) guidelines, funded by Moderna, Inc., and under the direction of the authors.

***Supplement sponsorship.*** This article appears as part of the supplement “COVID-19 Vaccination in the Immunocompromised Population,” sponsored by Moderna, Inc.
